# Claudins in genitourinary tract neoplasms: mechanisms, prognosis, and therapeutic prospects

**DOI:** 10.3389/fcell.2023.1308082

**Published:** 2023-12-22

**Authors:** Tarek Ziad Arabi, Nader Ashraf, Belal Nedal Sabbah, Abderrahman Ouban

**Affiliations:** ^1^ College of Medicine, Alfaisal University, Riyadh, Saudi Arabia; ^2^ Department of Pathology, College of Medicine, Alfaisal University, Riyadh, Saudi Arabia

**Keywords:** claudins, carcinoma, urothelial cell, prostate, renal cell carcinoma, treatment, prognosis, diagnosis

## Abstract

Genitourinary (GU) cancers are among the most prevalent neoplasms in the world, with bladder cancers constituting 3% of global cancer diagnoses. However, several pathogenetic mechanisms remain controversial and unclear. Claudins, for example, have been shown to play a significant role in several cancers of the human body. Their role in GU cancers has not been extensively studied. Aberrant expression of claudins −1, −2, −3, −4, −7, and −11 has been expressed in urothelial cell carcinomas. In prostate cancers, altered levels of claudins −1, −2, −3, −4, and −5 have been reported. Furthermore, the levels of claudins −1, −2, −3, −4, −6, −7, −8, and −10 have been studied in renal cell carcinomas. Specifically, claudins −7 and −8 have proven especially useful in differentiating between chromophobe renal cell carcinomas and oncocytomas. Several of these claudins also correlate with clinicopathologic parameters and prognosis in GU cancers. Although mechanisms underpinning aberrant expression of claudins in GU cancers are unclear, epigenetic changes, tumor necrosis factor-ɑ, and the p63 protein have been implicated. Claudins also provide therapeutic value through tailored immunotherapy via molecular subtyping and providing therapeutic targets, which have shown positive outcomes in preclinical studies. In this review, we aim to summarize the literature describing aberrant expression of claudins in urothelial, prostatic, and renal cell carcinomas. Then, we describe the mechanisms underlying these changes and the therapeutic value of claudins. Understanding the scope of claudins in GU cancers paves the way for several diagnostic, prognostic, and therapeutic innovations.

## 1 Introduction

**FIGURE 1:**
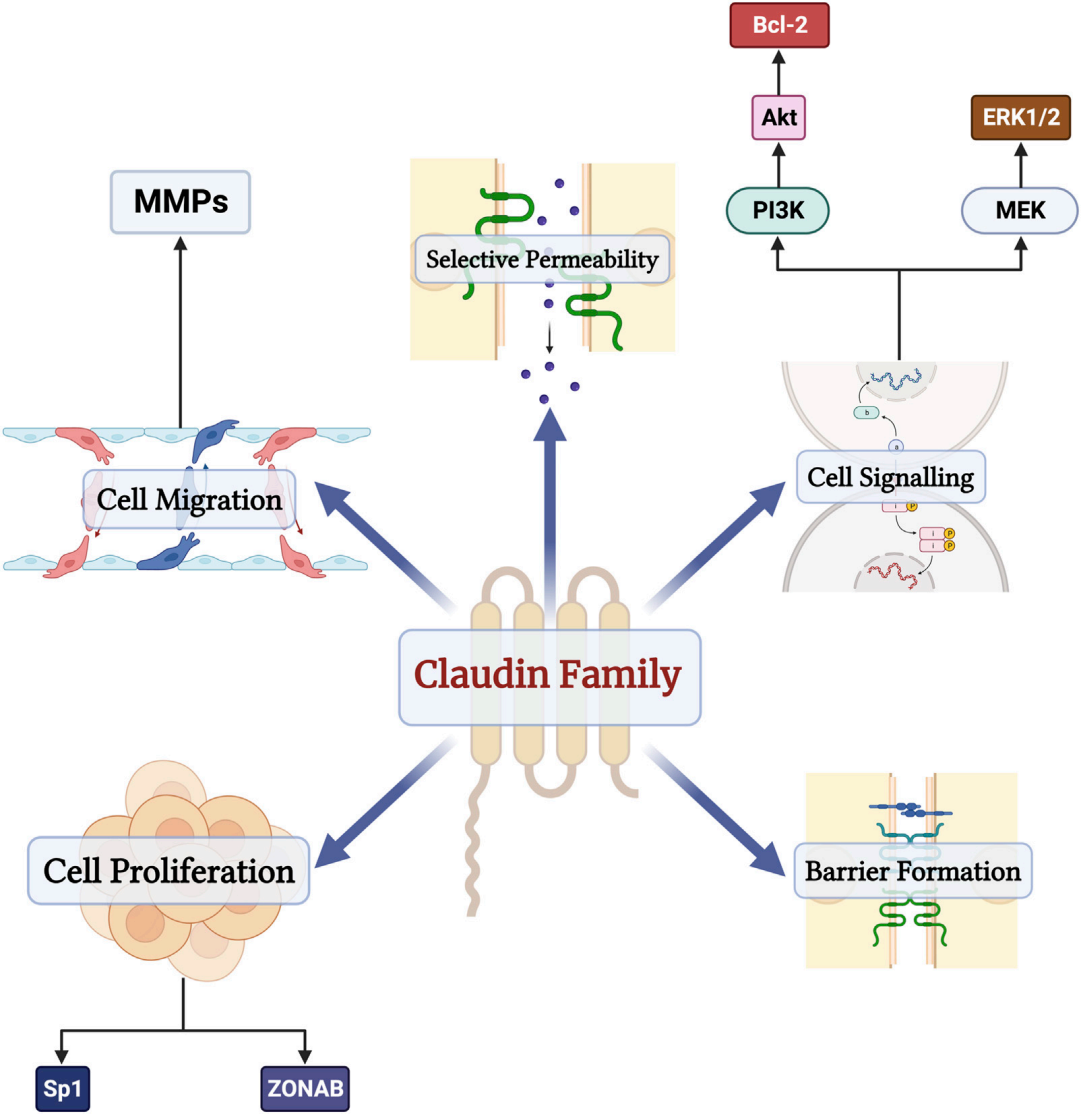
The physiologic functions of the claudin family of proteins in the human body. Besides their functions as barriers and channels, claudins also regulate cell proliferation through their interactions with transcription factors Sp1 and Zonula occludens 1-associated nucleic acid-binding protein (ZONAB), modulate cell migration through matrix metalloproteinases (MMPs), and serve as components of several signaling pathways such as MEK/ERK/1/2 and PI-3K/Bcl-2 pathways.

The barrier function of the epithelium is primarily maintained by tight junction proteins composed of zona-occludins, desmosomes, and claudins, among others (Arabi et al., 2023). The claudin family consists of 27 transmembrane proteins (Tsukita et al., 2019). Contrary to other tight junction proteins, some claudins function as paracellular channels and allow for the selective passage of water, anions, and cations (Günzel and Fromm, 2012). Besides their channel and barrier roles, claudins have several biologic effects (Figure 1). Claudins also play a role in cell migration through their effects on matrix metalloproteinases (MMP) (Venugopal et al., 2019). Furthermore, claudins, specifically claudin-2, also control the life cycle of cells and interact with several transcription factors, such as Sp1 and Zonula occludens 1-associated nucleic acid-binding protein (Venugopal et al., 2019). Claudins modulate several signaling pathways as well, including MEK/ERK/1/2 and PI-3K/Bcl-2 pathways (Ahmad et al., 2014; Venugopal et al., 2019). The broad spectrum of claudin functions is further solidified by claudin-knockout models, which lead to atopic dermatitis, renal hypoxia, seizures, and multiple sclerosis (Tsukita et al., 2019).

Emerging evidence has demonstrated that abnormal claudin expression can mediate tumorigenic processes in several cancers, including head and neck, gastrointestinal, breast, and lung neoplasms (Soini, 2011; Zhu et al., 2019; Yadav et al., 2022; Arabi et al., 2023). Additionally, aberrant expression of these proteins can be seen in preneoplastic conditions, highlighting their possible role for early detection and treatment of tumors (Ouban and Arabi, 2023).

Genitourinary (GU) cancers are among the most common cancers worldwide. For example, prostate cancers are the second most common solid tumors in men (Gandaglia et al., 2021). Bladder cancers constitute the 10th most common tumor worldwide, with 3% of all new cancer diagnoses (Bray et al., 2018). Unfortunately, due to higher rates of chemotherapy toxicity and comorbidities, elderly patients suffering from GU cancers often have poor cancer-related outcomes (Singhal et al., 2023). Hence, finding alternative treatment modalities may significantly increase the survival rates of this patient group.

Recent studies have shed some light on the role of claudins in GU cancers. For example, Săndulescu et al. demonstrated that claudin-4 expression is significantly increased in high-grade bladder urothelial carcinomas and those with invasion of the muscularis propria (SĂndulescu et al., 2020). Similarly, elevated levels of claudin-3 have been reported in prostatic cancer patients compared to controls (Ye et al., 2019). Reports of aberrant claudin expression and its role in the development of GU cancers remain limited. Moreover, the mechanisms underlying these changes remain unclear. Therefore, we will gather the latest literature surrounding aberrant expression of claudins and their prognostic implications in GU cancers, including bladder, prostate, and renal cell carcinomas (RCC). Then, we discuss the mechanisms mediating changes in claudin expression and their value as therapeutic targets.

## 2 Aberrant expression of claudins in urothelial carcinomas

Urothelial cells, previously known as transitional epithelial cells, line the lumen of the proximal urethra, bladder, ureters, and renal pelvis (Dalghi et al., 2020). The urothelium consists of several layers including the superficial umbrella cells, followed by the intermediate and basal layers, respectively (Dalghi et al., 2020). Urothelial carcinomas are seventh most common type of neoplasm and arises from the urothelium of the urethra, bladder, and ureters (Giudici et al., 2021). Bladder carcinomas are the most prevalent of urothelial carcinomas, constituting up to 95% of urothelial carcinomas (Giudici et al., 2021). Accordingly, the role of claudins in bladder carcinomas has been studied extensively. Claudin-1 can be detected at the membranes of high-grade urothelial carcinomas of the bladder (Săndulescu et al., 2022). However, a study by Elsen et al. found that both non-invasive bladder carcinoma and normal samples do not express claudin-1 (Elsen et al., 2015). Urothelial papillomas, papillary urothelial neoplasms of low malignant potential, and low-grade urothelial carcinomas of the bladder exhibit significantly less claudin-1 than inverted urothelial papillomas (Székely et al., 2011). Increased claudin-1 expression is also associated with better recurrence-free survival in papillary urothelial neoplasms of low malignant potential (Székely et al., 2011).

Claudin-2 can be seen as perimembranous cytoplasmic granules in the basal layers of nontumorous and tumorous urothelium (Törzsök et al., 2011). However, claudin-2 protein and messenger ribonucleic acid (mRNA) levels are increased in urothelial cell carcinomas compared to controls (Törzsök et al., 2011). Specifically, high-grade urothelial cell carcinomas express higher levels of claudin-2 mRNA compared to their low-grade counterpart (Törzsök et al., 2011). Claudin-3 is expressed in approximately 88% of urothelial carcinomas of the upper urinary tract on the upper epithelial layers (Nakanishi et al., 2008). Increased claudin-3 protein expression is associated with poor staging, grading, and pattern of tumor growth (Nakanishi et al., 2008). Although claudin-3 expression is correlated with overall survival rates in univariate analysis, multivariate analysis has failed to demonstrate a similar effect (Nakanishi et al., 2008).

A study by Chen et al. aimed to assess the alterations of tight junction proteins in bladder carcinoma cells compared to adjacent non-neoplastic tissue (Chen et al., 2020). The authors demonstrated elevated expression of claudin-4 in neoplastic tissue (Chen et al., 2020). However, claudin-4 levels are not associated muscle invasion, lymph node metastasis, or distant metastasis (Chen et al., 2020). Specifically, claudin-4 is overexpressed in low/medium-grade bladder carcinomas and becomes underexpressed in high-grade tumors (Boireau et al., 2007). Alterations in claudin-4 expression have been linked to hypermethylation, with inhibition of methylation enzymes promoting increases in cell polarization and transepithelial resistance (Boireau et al., 2007). Claudin-4 expression has also been studied in upper urinary tract carcinomas (Nakanishi et al., 2008). The protein is localized to the plasma membranes of neoplastic cells and is present in around three-quarters of tumor biopsies. Although claudin-4 has been linked to poor staging, no effect has been reported on disease-free and overall survival in this patient population (Nakanishi et al., 2008).

Gadelmoula et al. studied the expression of claudin-7 proteins and mRNA in urothelial bladder carcinoma patients (Gadelmoula et al., 2013). The authors demonstrated that claudin-7 is significantly downregulated compared to control specimens. Muscle-invasive tumors also exhibit less claudin-7 than their non-invasive counterparts; however, there was no difference in claudin-7 expression between different tumor grades (Gadelmoula et al., 2013). Then, Yamuç et al. examined the correlation between claudin-7 and patient outcomes (Yamuç et al., 2022). Although claudin-7 levels were different between genders, tumor stage, grade, and gender had no impact on the protein. Nevertheless, loss of claudin-7 was associated with tumor recurrence and poor overall survival. Contrarily, the utility of claudin-7 to differentiate between the histological grade of urothelial carcinomas has been reported, where high-grade tumors exhibit lower levels than low-grade ones (Törzsök et al., 2011). Claudin-7 is not associated with clinicopathologic factors, recurrence-free, or overall survival in urothelial carcinomas of the upper urinary tract (Nakanishi et al., 2008). The direct impact of claudin-7 on urothelial carcinomas is controversial; however, claudin-7 appears to play an antineoplastic role in this group of tumors. Further studies are needed to confirm the prognostic role of claudin-7 in urothelial carcinoma patients.

Awsare et al. studied the role of claudin-11 in human bladder carcinomas (Awsare et al., 2011). Claudin-11 mRNA levels were not different between carcinogenic and control samples; however, claudin-11 staining was less in the tumor samples when using immunohistochemistry. Forced expression of claudin-11 in bladder cancer cell lines blunted tumor invasion and increased cell matrix adhesion but increases tumor growth as well. Overall, alterations in the expression of claudins −1, −2, −3, −4, −7, and −11 have been reported in urothelial carcinomas (Table 1). Some of these claudins have shown prognostic value (Table 2), although further research is needed to validify these findings and clarify any contradictions.

**TABLE 1:** The changes of claudin levels in various neoplasms of the genitourinary tract.

Location	Type of tumor	Claudin isoform	Level of expression	Type of measured marker	References
Urinary Tract	Urothelial carcinoma of the lower tract	Claudin-1	↑^a^, ↓^b^	Protein	Székely et al. (2011), Săndulescu et al. (2022)
		Claudin-2	↑^a^	Protein and mRNA	Törzsök et al. (2011)
		Claudin-4	↓^a^, ↑^b^	Protein and mRNA	Boireau et al. (2007)
		Claudin-7	↓	Protein and mRNA	Gadelmoula et al. (2013)
		Claudin-11	↓	Protein and mRNA	Awsare et al. (2011)
	Urothelial papillomas	Claudin-1	↓	Protein	Székely et al. (2011)
	Papillary urothelial neoplasms of low malignant potential	Claudin-1	↓	Protein	Székely et al. (2011)
	Urothelial carcinoma of the upper tract	Claudin-3	↑	Protein	Nakanishi et al. (2008)
		Claudin-4	↑	Protein	Nakanishi et al. (2008)
Prostate	Prostatic adenocarcinoma	Claudin-1	↓	Protein	Sheehan et al. (2007), Kind et al. (2020)
		Claudin-2	↑	Protein	Coutinho-Camillo et al. (2011)
		Claudin-3	↑; ↓^c^	Protein	Coutinho-Camillo et al. (2011), Worst et al. (2017), Ye et al. (2019)
		Claudin-4	↑	Protein	Radi and Abd-Elazeem (2016)
		Claudin-5	↑; ↓^d^	Protein	Coutinho-Camillo et al. (2011)
		Claudin-7	↓^d^	Protein	Sheehan et al. (2007)
		Claudin-8	↑^d^	Protein and mRNA	Ashikari et al. (2017)
Kidney	Clear cell RCC	Claudin-1	↓	Protein	Fritzsche et al. (2008), Shin et al. (2011)
		Claudin-4	↓	Protein	Lechpammer et al. (2008)
		Claudin-8	↓	Protein and mRNA	Zhu et al. (2020)
		Claudin-10	↓	Protein and mRNA	Yang et al. (2021), Yang et al. (2022)
	Papillary RCC	Claudin-6	↓	Protein	Mikuteit et al. (2022)
	Chromophobe RCC	Claudin-6	↓	Protein	Erlmeier et al. (2023)
		Claudin-7	↑	Protein	Lechpammer et al. (2008), Li et al. (2008), Gutiérrez et al. (2018)
	Unspecified RCC	Claudin-2	↓	Protein and RNA	Kumar et al. (2021)

^a^ High-grade.

^b^ Low-grade.

^c^ CRPC.

^d^ Conflicting data.

**TABLE 2:** The prognostic and diagnostic significance of claudins in genitourinary tract neoplasms.

Location	Type of tumor	Claudin isoform	Diagnostic/prognostic significance	References
Urinary tract	Urothelial carcinomas of the lower tract	Claudin-7	Tumor recurrence and poor survival	Gadelmoula et al. (2013)
	Papillary urothelial neoplasms of low malignant potential	Claudin-1	Poor recurrence-free survival	Székely et al. (2011)
	Urothelial carcinoma of the upper tract	Claudin-3	Poor staging, grading, and pattern of growth	Nakanishi et al. (2008)
Prostate	Prostatic adenocarcinoma	Claudin-1	Reduced risk of PSA recurrence^a^	Sheehan et al. (2007), Kind et al. (2020)
		Claudin-3	Distant metastasis; poor disease-free survival and time to progression^b^	Coutinho-Camillo et al. (2011), Worst et al. (2017), Ye et al. (2019)
		Claudin-4	Lymph node metastasis	Radi and Abd-Elazeem (2016)
Kidney	Clear cell RCC	Claudin-1	Increased levels associated with metastasis	Fritzsche et al. (2008), Shin et al. (2011)
		Claudin-8	Poor overall survival	Zhu et al. (2020)
		Claudin-10	Poor disease-free and overall survival	Yang et al. (2021), Yang et al. (2022)
	Papillary RCC	Claudin-1	Increased levels compared to chromophobe RCC	Lechpammer et al. (2008)
	Chromophobe RCC	Claudin-7	Differentiates between other subtypes with 95% sensitivity and 92.3% specificity	Choi et al. (2007), Zhou et al. (2019)
			Differentiates between papillary RCC, clear cell RCC, and oncocytomas with 94% sensitivity and 78% specificity, which increases to 100% when combining other markers	
		Claudin-8	Decreased levels compared to oncocytomas	Lechpammer et al. (2008), Kim et al. (2009), Osunkoya et al. (2009)
	Unspecified RCC	Claudin-2	Progression and poor survival	Kumar et al. (2021)

^a^ Conflicting data.

^b^ CRPC.

## 3 Aberrant expression of claudins in prostate cancer

After analyzing over 17,000 prostate cancer specimens, Kind et al. found claudin-1 protein in 39% of cases, suggesting upregulation during malignant transformation compared to normal glandular cells (Kind et al., 2020). Two other studies, with smaller cohorts of 101 and 141 cases, also reported claudin-1 upregulation in 97% and 41% of tumors relative to normal prostatic glands (Sheehan et al., 2007; Väre et al., 2008). The differences in positive rates between these three studies likely stem from variations in staining protocols and evaluation criteria.

While claudin-1 expression is found normally in the prostate, there is conflicting data regarding its clinical utility. Four studies involving a total of 30, 48, 101, and 141 specimens have reported that the downregulation of claudin-1 is linked to unfavorable tumor characteristics, a poor prognosis, and/or biochemical recurrence (Sheehan et al., 2007; Väre et al., 2008; Seo et al., 2010; Szász et al., 2010). In fact, through multivariate analysis, one study identified reduced claudin-1 expression as an independent predictor of tumor recurrence (Sheehan et al., 2007). Consistent with previous studies, Kind et al. found that claudin-1 upregulation is associated with favorable tumor features, low Gleason grade, and a reduced risk of PSA recurrence (Kind et al., 2020). Particularly, this was most relevant in ERG-positive prostate cancer where increased claudin-1 expression predicts a favorable prognosis (Kind et al., 2020). Nonetheless, multivariate analyses did not reveal an independent association of claudin-1 expression with prognosis (Kind et al., 2020).

However, these findings contradict the research by Krajevska et al. who reported claudin-1 expression in the basal cells of normal prostate glands, but its complete absence in luminal/glandular cells and in 98% of the analyzed cancer samples (Krajewska et al., 2007). This characteristic staining pattern of claudin-1 in the normal prostate was also described by Kind et al. (Kind et al., 2020). It is hypothesized that the loss of claudin-1 expression in prostate cancer could potentially indicate epithelial-mesenchymal transition (Medici et al., 2006; Yang et al., 2006).

In a study exploring the connection between abnormal expressions of claudin-1, claudin-3, claudin-4, and claudin-7 and prognostic factors in prostate cancer, Sheehan et al. determined that, like claudin-1, reduced claudin-7 expression correlated with poorly differentiated histological tumors (Sheehan et al., 2007). Conversely, they found that elevated levels of claudin-3 and claudin-4 were linked to advanced-stage tumors (Sheehan et al., 2007). In contrast, Camillo et al. demonstrated claudins-1 and -7 were equivocal, claudins-2, -3, and -5 were overexpressed, and claudin-4 was downregulated in prostate adenocarcinomas compared with normal samples (Coutinho-Camillo et al., 2011). However, even with all these alterations in claudin levels, they did not find any association with the main clinicopathological parameters.

Similar findings were observed by other studies regarding elevated claudin-3 and higher Gleason grade (Coutinho-Camillo et al., 2011; Worst et al., 2017). Specifically, Ye et al. found claudin-3 levels to be associated with distant metastasis in cancer cells, preoperative PSA levels, tumor diameter, and the pathological stage of the disease (Ye et al., 2019). Interestingly, in castration-resistant prostate cancer (CRPC), claudin-3 expression significantly decreased in samples from patients with high total Gleason scores (≥8) and locally advanced tumors, and this loss of expression was associated with worse disease-free survival and time to clinical progression (Orea et al., 2023). These findings strongly suggest that epigenetic silencing of claudin-3 is a common occurrence in CRPC, potentially serving as a valuable molecular marker for predicting the prognosis of prostate cancer patients and differentiating aggressive from indolent prostate tumors.

The observation by Sheehan et al. (Sheehan et al., 2007) regarding claudin-4 was similar to other studies (Landers et al., 2008; Väre et al., 2008; Szász et al., 2010; Radi and Abd-Elazeem, 2016). One of those studies highlighted high claudin-4 expression as a poor prognosis biomarker due to its association with high tumor grade, lymphovascular invasion, positive lymph node metastasis, and high mean peritumoral lymphatic vessel density (Radi and Abd-Elazeem, 2016). Furthermore, Landers et al. found heightened expression in prostate cancer and metastatic prostate carcinoma compared to prostatic hyperplasia tissues (Landers et al., 2008). One noteworthy difference from the report of Sheehan et al. (Sheehan et al., 2007), Landers et al.’s immunohistochemical analysis of claudin-4 was supported by a comparative evaluation of α-Methylacyl-CoA racemase (AMACR) and prostate-specific membrane antigen (PSMA)—two well-established prostate cancer markers (Landers et al., 2008).

When examining sections containing high-grade prostatic intraepithelial neoplasia (HG-PIN) without associated invasive carcinoma, Landers et al. observed moderate claudin-4 intensity and low PSMA and AMACR levels (Landers et al., 2008). In contrast, HG-PIN cells in conjunction with invasive carcinoma displayed strong claudin-4 staining, but AMACR levels were low and PSMA levels were moderate (Landers et al., 2008). This suggests that claudin-4 may play a role in the early stages of prostate cancer development. The authors also discovered that lower-grade carcinomas exhibit higher claudin-4 expression compared to higher-grade carcinomas, with epithelial cells in benign glands surrounding the cancer sections displaying moderate to strong claudin-4 staining (Landers et al., 2008). This is consistent with loss of cellular organization as cancer progresses leading to a reduction in tight junction function, which aligns with alterations cellular polarity that enhance cellular mobility (Weinstein et al., 1976). Moreover, claudin-4 staining was strongly positive in most metastatic tumors, with no noticeable difference between specimens from patients receiving androgen suppression and those who were not (Landers et al., 2008). Also, there seemed to be no correlation between the metastatic site and the intensity of claudin-4 expression.

Generally, claudin-5 was found to be weakly expressed in prostate cancer compared to normal tissue, particularly in clinically poorly behaving carcinomas (Väre et al., 2008; Seo et al., 2010; Szász et al., 2010). As for claudins −7 and −10, studies showed equivocal results (Väre et al., 2008; Szász et al., 2010; Coutinho-Camillo et al., 2011). Nonetheless, one study found an association between low expression of claudin-7 and higher tumor grade (Sheehan et al., 2007). To note, two forms of claudin-7 exist, a full-length form of CLDN-7 with 211 amino-acid residues and a C-terminal truncated form with 158 amino-acid residues, both of which were found to regulate the expression of PSA (Zheng et al., 2003). Hence, it can be of interest for future studies to explore the difference in expression of both forms of claudin-7 and correlate it to prognostic markers.

While Szász et al. observed a significant decrease in claudin-8 expression, it did not have any clinical correlation (Szász et al., 2010). On the contrary, Ashikari et al. indicated that claudin 8 was overexpressed in prostate cancer clinical samples compared to benign tissues (Ashikari et al., 2017). Moreover, their findings demonstrate that claudin-8 not only regulates intracellular signal transduction and stabilizes the cytoskeleton but also operates as an androgen receptor downstream signal, collectively contributing to the advancement of prostate cancer. Collectively, several claudins are altered in prostate cancers, among which several have clinical significance (Table 1).

## 4 Aberrant expression of claudins in renal cell carcinomas

Kidney neoplasms are the sixth and 10th most common cancers in men and women, respectively (Bahadoram et al., 2022). RCC, the most common type of renal tumors, constitutes approximately 3% of all adult malignancies (Rini et al., 2009). RCCs are generally divided into five subtypes: clear cell, papillary, chromophobe, collecting duct, and unclassified, among which the first three constitute the majority of RCCs (Truong and Shen, 2011). Although advances in the medical and surgical management of RCCs have been made (Rini et al., 2009; Bazarbashi et al., 2023), the outcomes associated with the tumor remain relatively poor (Badran et al., 2020). Additionally, differentiating between different subtypes of RCC is still difficult. Claudins have been used to fill these gaps as diagnostic, prognostic, and therapeutic tools in RCCs. In this section, we will summarize the evidence discussing these applications (Table 1).

Claudin-1 is expressed in renal biopsies as a membranous pattern, mostly in the Bowman membrane and, to a lesser degree, the epithelium of the distal tubules and collecting ducts (Fritzsche et al., 2008; Lechpammer et al., 2008). On the other hand, the protein is only seen in one-third of RCC samples (Fritzsche et al., 2008). Claudin-1 mainly localizes at the plasma membrane and sometimes in the cytoplasm of neoplastic cells. Specifically, three-quarters of papillary RCCs express claudin-1, while only a quarter of clear cell RCCs exhibit the protein (Fritzsche et al., 2008). In clear cell RCC, claudin-1 expression is associated with metastasis and high-grade tumors (Fritzsche et al., 2008; Shin et al., 2011). Claudin-1 expression correlates with poor disease-specific survival only in asymptomatic clear cell RCC patients but not in all patients (Fritzsche et al., 2008). However, the protein is associated with increased postoperative distant metastasis in clear cell RCC patients (Shin et al., 2011). Contrarily, claudin-1 expression is associated with improved clinicopathologic characteristics in papillary RCC, but not survival (Fritzsche et al., 2008). Claudin-1 immunohistochemistry can also be used to differentiate papillary RCC from its chromophobe counterpart, which exhibits weak expression (Lechpammer et al., 2008).

Claudin-2 is significantly reduced in RCC samples, and loss of the protein is associated with tumor progression and poor survival (Kumar et al., 2021). Claudin-2 overexpression inhibits mesenchymal plasticity, tumorigenic abilities, and tumor growth in murine models (Kumar et al., 2021). Claudin-3 is strongly expressed in the distal tubules and collecting ducts and weakly in the glomeruli and proximal tubules of normal renal samples (Lechpammer et al., 2008). Claudin-3 expression varies from moderate to strong in papillary, chromophobe, and clear cell RCCs (Lechpammer et al., 2008). Claudin-3 is predominantly seen in lower grades of clear cell RCCs and is not associated with tumor grade in other subtypes (Lechpammer et al., 2008). Although claudin-3 is associated with poor survival in univariate analysis, multivariate analysis diminishes this effect (Lechpammer et al., 2008).

Strong expression of claudin-4 in normal renal samples can be seen in the cell membrane and cytoplasm of distal tubules and collecting ducts cells (Lechpammer et al., 2008; Owari et al., 2020). Claudin-4 is not associated with histological grade or staging in RCC patients (Owari et al., 2020). Chromophobe RCCs also exhibit moderate to strong claudin-4 expression, while only half of clear cell RCCs express the protein (Lechpammer et al., 2008). Similar to claudin-3, strong expression of claudin-4 is associated with poor survival in clear cell RCC using univariate but not multivariate analysis (Lechpammer et al., 2008).

Studies reporting the presence of claudin-6 in RCCs are limited. Mikuteit et al. assessed the prognostic role of the protein in papillary RCCs (Mikuteit et al., 2022). Claudin-6 was present in around one-fifth of patients with type 1 and type 2 papillary RCC. Expression of the protein had no impact on survival in these patients. Then, Erlmeier et al. aimed to assess the same in chromophobe RCC patients (Erlmeier et al., 2023). Claudin-6 was only seen in 12.3% of chromophobe tumor samples. Like papillary RCC, claudin-6 expression was not correlated with overall survival (Erlmeier et al., 2023). To the best of the authors’ knowledge, these are the only available reports of claudin-6 expression in RCC samples. Future studies should aim to define its role in clear cell RCCs and other subtypes.

In normal renal samples, claudin-7 is only expressed in the distal convoluted tubules and distal collecting ducts (Lechpammer et al., 2008). Membranous claudin-7 expression can be seen in the majority of chromophobe and, to a lesser extent, papillary RCCs (Lechpammer et al., 2008; Li et al., 2008; Gutiérrez et al., 2018). Claudin-7 has proven useful as a diagnostic tool to differentiate between different renal tumors, especially oncocytomas and chromophobe RCC (Osunkoya et al., 2009; Ohe et al., 2012). The protein has a sensitivity and specificity of 95% and 92.3% in discriminating between chromophobe RCC and other RCC subtypes (Choi et al., 2007). When differentiating chromophobe tumors from papillary RCCs, clear cell RCCs, and oncocytoma, the protein demonstrates a sensitivity and specificity of 94% and 78%, respectively (Zhou et al., 2019). Specificity can be further increased to 100% when combining claudin-7 with additional markers CD117 and CK7 (Zhou et al., 2019).

The prognostic implications of claudin-7 expression are controversial. In a study of 120 clear cell RCC patients, reduced claudin-7 expression predicted poor staging and survival (Li et al., 2018). In line with these findings, forced overexpression of claudin-7 triggers neoplastic cell apoptosis, blunts proliferation, and inhibits tumor invasion and migration (Li et al., 2018). However, another study by Shin et al. found no association between the protein with disease-free and overall survival (Shin et al., 2011). Despite the clear diagnostic practicality of using claudin-7, more investigations are needed into its prognostic role.

Similar to claudin-7, claudin-8 exhibits membranous staining patterns in the distal tubules and collecting ducts (Lechpammer et al., 2008). Clear cell RCCs exhibit lower claudin-8 mRNA and protein levels than normal tissue samples (Zhu et al., 2020). The protein appears to play a role in facilitating pro-neoplastic effects. Zhu et al. studied the effects of claudin-8 on clear cell RCC progression and its prognostic value (Zhu et al., 2020). Claudin overexpression attenuated tumor invasion, migration, and proliferation *via* its modulatory effects on epithelial-to-mesenchymal transition and Akt pathways (Zhu et al., 2020). Low claudin-8 levels were also independently associated with poor overall survival in their group of patients (Zhu et al., 2020). Furthermore, chromophobe RCCs demonstrate significantly reduced cytoplasmic staining of claudin-8 than oncocytomas, highlighting its diagnostic utility (Lechpammer et al., 2008; Kim et al., 2009; Osunkoya et al., 2009). Whether claudin-8 has any prognostic value in other RCC subtypes remains unstudied.

Claudin-10 expression is significantly blunted in clear cell RCC (Yang et al., 2021). Increased claudin-10 mRNA expression is independently associated with longer disease-free and overall survival in clear cell RCC patients (Yang et al., 2021; Yang et al., 2022). Claudin-10 overexpression induces apoptosis and inhibits migration, invasion, and proliferation by promoting mitochondrial dysfunction (Yang et al., 2022). Moreover, claudin-10 is associated with increased dendritic cell and naïve and memory T-cell tumor infiltration, which has been linked with better outcomes (Yang et al., 2021). Aberrant expression of claudins −14, −17, −19, and −22 has also been reported in RCCs (Men et al., 2015; Singh and Vinod, 2020); however, reports about these proteins are still extremely limited.

## 5 Mechanisms causing aberrant expression of claudins in GU cancers

**FIGURE 2:**
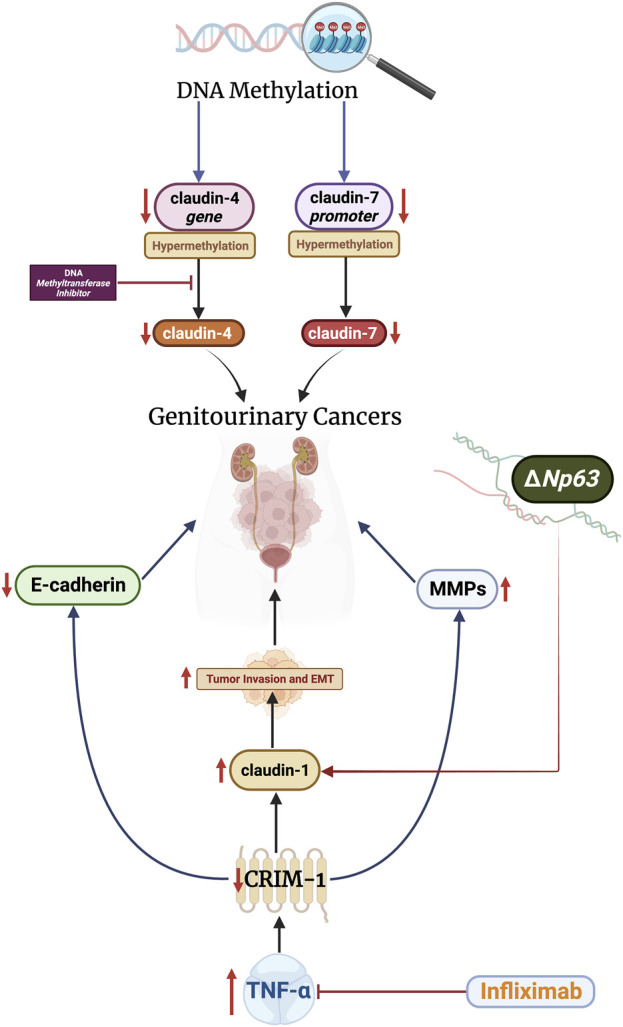
Alterations relating to ΔNp63 and CRIM-1 increase claudin-1 levels, which promote tumor invasion and epithelial-mesenchymal transition (EMT). Claudins-4 and -7 are modulated by DNA methylation. These mechanisms can be targeted therapeutically through DNA methyltransferase inhibitors and infliximab, which have shown promising results.

The mechanisms underlying aberrant expression of claudins in GU carcinomas are not completely understood. Nevertheless, several mechanisms have been studied and reported (Figure 2). Epigenetic modifications–mainly through DNA methylation–appear to play a crucial role in the induction of aberrant expression. For example, the claudin-4 gene is hypermethylated in bladder carcinomas (Boireau et al., 2007). Treatment with DNA methyltransferase inhibitors reverses the effects of *CLDN4* methylation, increasing cell polarization and transepithelial resistance (Boireau et al., 2007). In RCC, promoter hypermethylation is associated with reduced claudin-7 mRNA and protein expression (Li et al., 2018). Hypermethylation of the claudin-7 promoter is associated with advanced tumors and poor survival in these patients (Li et al., 2018). Furthermore, hypermethylation of claudin −8, −9, −10, −14, −19, and −22 promoters has been reported in papillary RCC (Singh and Vinod, 2020).

Transmembrane proteins, specifically cysteine-rich motor neuron-1 (CRIM-1), also regulate the expression of claudins in GU cancers. The CRIM-1 protein is highly expressed in the kidney and placenta cells (Kolle et al., 2000). Ogasawara et al. analyzed the effects of CRIM-1 on proliferation, malignant transformation, and claudin expression in RCCs (Ogasawara et al., 2018). The authors demonstrated that decreased expression of CRIM-1 does not impact tumor growth or proliferation. However, the authors revealed that under-expression of CRIM-1 increases tumor invasion and epithelial-mesenchymal transition by reducing E-cadherin expression and increasing claudin-1 and MMP-2 and -9 expression. Expression of CRIM-1 was blunted by tumor necrosis factor (TNF)-ɑ, indicating a potential therapeutic value of targeting this pathway. In line with this, treatment with infliximab, an anti-TNF-ɑ antibody, resulted in 16% of RCC patients achieving partial response and 16% achieving stable disease in a phase II trial (Harrison et al., 2007). Nevertheless, the efficacy of infliximab in RCC remains controversial and further studies are still needed (Larkin et al., 2010).

The p63 gene, a member of the tumor suppressor p53 family, has two different promotes which produce isotypes that contain or lack (ΔN) an NH2-terminus (Chen et al., 2018). Studies have demonstrated the regulatory effects of p63 on claudin expression in epithelial cells (Kojima et al., 2017). However, this relationship remains unclear in GU cancers. The ΔNp63 isotype is overexpressed in bladder cell carcinomas (Park et al., 2000; Jing et al., 2013). In bladder carcinoma cell lines, ΔNp63 promotes tumor invasion and metastasis through overexpression of claudin-1, and knockdown of the isotype blunts these effects (Jing et al., 2013). Conclusively, epigenetics, transmembrane proteins, and the p63 gene modulate claudin expression in various GU cancers. Studies are still limited in this regard and more investigations are needed to implicate additional signaling pathways.

## 6 The therapeutic value of claudins in GU cancers

Several therapeutic avenues targeting claudins have been explored in the context of GU carcinomas. Firstly, claudin expression has been used to tailor management and predict the response of patients to certain drugs. Despite claudin-low bladder tumors expressing high levels of immune gene signatures, they also exhibit high levels of immune checkpoint molecules (Kardos et al., 2016). These findings have led some to hypothesize that molecular subtypes can be used to predict immunotherapeutic responses (Kardos et al., 2016). In line with these findings, the PURE-01 study assessed the effects of preoperative pembrolizumab on post-cystectomy remission times. The study revealed that claudin-low muscle-invasive bladder cancer patients exhibit longer event-free survival than other patient categories after pembrolizumab and radical cystectomy (Bandini et al., 2020; Necchi et al., 2020).

Antibodies targeting different claudins have also begun emerging. Among them, Kuwada et al. developed a rat antibody targeting the extracellular domain of the claudin-4 protein (Kuwada et al., 2015). The antibody inhibited bladder cell growth and invasion and promoted apoptosis (Kuwada et al., 2015). Furthermore, the antibody increased cellular permeability to cisplatin, inhibiting tumor growth and improving survival in mice models (Kuwada et al., 2015).

Bufalin–a steroid obtained from toads–exhibits antineoplastic effects in several tumors, such as gastrointestinal, breast, and lung cancers (Jiang et al., 2010; Takai et al., 2012). Bufalin inhibits bladder cancer migration and invasion by reducing expression of several claudins and MMPs, possibly through phosphorylation of the extracellular signal-regulated protein kinase (ERK) pathway (Hong et al., 2013). Diallyl trisulfide–a compound extracted from garlic–also exhibits antineoplastic similar effects to bufalin in bladder cancer cell lines (Shin et al., 2013). Finally, the *Clostridium perfringens* enterotoxin (CPE), which binds to claudins −3 and −4, induces cell death in low and moderately aggressive bladder cancer cells (Gabig et al., 2016). The non-toxic fragment of CPE also increases cell sensitivity to mitomycin C and dasatinib (Gabig et al., 2016). Claudins have provided promising therapeutic value in both clinical and preclinical studies. Nevertheless, human studies are needed to determine whether direct targeting of claudins is impactful in clinical settings.

## 7 Conclusion and future perspectives

GU cancers are among the most prevalent neoplasms worldwide. Despite several advances in diagnosis and therapeutics, poor outcomes are seen in a large portion of patients. Claudins have piqued the interest of the scientific community due to the aberrant expression seen in a wide spectrum of tumors. Several studies have expanded on this in the context of GU cancers, such as those in the bladder, prostate, and kidneys. Abnormal expression of claudins, such as claudins −1 and −7, has been linked to clinicopathological characteristics and prognosis in GU cancer patients. Furthermore, claudins have been used to histologically differentiate between different types of renal tumors, specifically chromophobe RCCs and oncocytomas.

This manuscript also summarizes the different pathways involved in promoting aberrant claudin expression. DNA methylation impacts the expression of various claudins, and such effects have been reported in several types of GU tumors. TNF-ɑ modulates the expression of claudin-1 and promotes tumor invasion. Finally, the ΔNp63 isotype exerts pro-neoplastic effects by increasing claudin-1 expression in bladder cancer cells.

In the realm of claudin research within GU neoplasms, there lies a fertile ground for future research, particularly in unraveling the molecular mechanisms of aberrant expression of claudins. The interactions of claudins with other tight junction proteins and their roles in cellular signaling pathways are pivotal areas that demand deeper investigation, especially concerning their contribution to tumorigenesis and metastasis. With the advent of cutting-edge technologies such as CRISPR/Cas9 gene editing and advanced imaging, new dimensions of claudin behavior in cancer cells can be unveiled, potentially leading to more accurate disease models and real-time visualization of tumor progression. Molecular targeted magnetic resonance imaging is capable of detecting claudin-2 in interstitial cystitis mouse models (Smith et al., 2020). Similar imaging techniques could be utilized in the future to accurately detect and track claudin-positive GU neoplasms.

The field also grapples with unresolved questions, notably the heterogeneity of claudin expression in various cancer subtypes and stages, and its implications on prognosis and therapeutic response. The exploration of claudin-based therapeutics, despite its promising outlook, remains in its nascent stage, requiring more rigorous clinical trials and research to ascertain its efficacy and safety. Looking ahead, the integration of claudins into personalized medicine emerges as a critical frontier. Developing diagnostic tools and treatment strategies based on individual claudin profiles could revolutionize patient care. Additionally, the synergy of claudin-targeted therapies with established treatment modalities like chemotherapy, immunotherapy, or radiation presents a promising avenue for enhancing treatment outcomes and overcoming resistance.
